# Prognostic role of radiomics‐based body composition analysis for the 1‐year survival for hepatocellular carcinoma patients

**DOI:** 10.1002/jcsm.13315

**Published:** 2023-08-17

**Authors:** Sylvia Saalfeld, Robert Kreher, Georg Hille, Uli Niemann, Mattes Hinnerichs, Osman Öcal, Kerstin Schütte, Christoph J. Zech, Christian Loewe, Otto van Delden, Vincent Vandecaveye, Chris Verslype, Bernhard Gebauer, Christian Sengel, Irene Bargellini, Roberto Iezzi, Thomas Berg, Heinz J. Klümpen, Julia Benckert, Antonio Gasbarrini, Holger Amthauer, Bruno Sangro, Peter Malfertheiner, Bernhard Preim, Jens Ricke, Max Seidensticker, Maciej Pech, Alexey Surov

**Affiliations:** ^1^ Research Campus STIMULATE at the University of Magdeburg Magdeburg Germany; ^2^ Department of Simulation and Graphics University of Magdeburg Magdeburg Germany; ^3^ University Library University of Magdeburg Magdeburg Germany; ^4^ Department of Radiology and Nuclear Medicine OvGU Magdeburg Magdeburg Germany; ^5^ Department of Radiology LMU University Hospital Munich Germany; ^6^ Department of Internal Medicine and Gastroenterology Niels‐Stensen‐Kliniken Marienhospital Osnabrück Germany; ^7^ Klinik für Gastroenterologie, Hepatologie und Endokrinologie Medizinische Hochschule Hannover (MHH) Hannover Germany; ^8^ Department of Radiology and Nuclear Medicine University Hospital Basel, University of Basel Basel Switzerland; ^9^ Section of Cardiovascular and Interventional Radiology, Department of Bioimaging and Image‐Guided Therapy Medical University of Vienna Vienna Austria; ^10^ Department of Radiology and Nuclear Medicine Academic University Medical Centers Amsterdam The Netherlands; ^11^ Department of Radiology University Hospitals Leuven Leuven Belgium; ^12^ Department of Digestive Oncology University Hospitals Leuven Leuven Belgium; ^13^ Department of Radiology Charité – University Medicine Berlin Berlin Germany; ^14^ Department of Radiology Grenoble University Hospital La Tronche France; ^15^ Diagnostic and Interventional Radiology Candiolo Cancer Institute Turin Italy; ^16^ Fondazione Policlinico Universitario A. Gemelli IRCCS, UOC di Radiologia d'Urgenza e Interventistica, Dipartimento di Diagnostica per Immagini, Radioterapia Oncologica ed Ematologia Rome Italy; ^17^ Università Cattolica del Sacro Cuore Rome Italy; ^18^ Klinik und Poliklinik für Gastroenterologie, Sektion Hepatologie Universitätsklinikum Leipzig Leipzig Germany; ^19^ Department of Medical Oncology Amsterdam University Medical Centers Amsterdam The Netherlands; ^20^ Department of Hepatology and Gastroenterology Campus Virchow Klinikum, Charité – Universitätsmedizin Berlin Berlin Germany; ^21^ Fondazione Policlinico Universitario Gemelli IRCCS, Università Cattolica del Sacro Cuore Rome Italy; ^22^ Department of Nuclear Medicine Charité – Universitätsmedizin Berlin, corporate member of Freie Universität Berlin and Humboldt Universität zu Berlin Berlin Germany; ^23^ Liver Unit Clínica Universidad de Navarra and CIBEREHD Pamplona Spain; ^24^ Department of Medicine II University Hospital, LMU Munich Munich Germany; ^25^ Department of Radiology, Neuroradiology and Nuclear Medicine Johannes Wesling University Hospital, Ruhr University Bochum Bochum Germany

**Keywords:** body composition, HCC, radiomics, sarcopenia

## Abstract

**Background:**

Parameters of body composition have prognostic potential in patients with oncologic diseases. The aim of the present study was to analyse the prognostic potential of radiomics‐based parameters of the skeletal musculature and adipose tissues in patients with advanced hepatocellular carcinoma (HCC).

**Methods:**

Radiomics features were extracted from a cohort of 297 HCC patients as post hoc sub‐study of the SORAMIC randomized controlled trial. Patients were treated with selective internal radiation therapy (SIRT) in combination with sorafenib or with sorafenib alone yielding two groups: (1) sorafenib monotherapy (*n* = 147) and (2) sorafenib and SIRT (*n* = 150). The main outcome was 1‐year survival. Segmentation of muscle tissue and adipose tissue was used to retrieve 881 features. Correlation analysis and feature cleansing yielded 292 features for each patient group and each tissue type. We combined 9 feature selection methods with 10 feature set compositions to build 90 feature sets. We used 11 classifiers to build 990 models. We subdivided the patient groups into a train and validation cohort and a test cohort, that is, one third of the patient groups.

**Results:**

We used the train and validation set to identify the best feature selection and classification model and applied it to the test set for each patient group. Classification yields for patients who underwent sorafenib monotherapy an accuracy of 75.51% and area under the curve (AUC) of 0.7576 (95% confidence interval [CI]: 0.6376–0.8776). For patients who underwent treatment with SIRT and sorafenib, results are accuracy = 78.00% and AUC = 0.8032 (95% CI: 0.6930–0.9134).

**Conclusions:**

Parameters of radiomics‐based analysis of the skeletal musculature and adipose tissue predict 1‐year survival in patients with advanced HCC. The prognostic value of radiomics‐based parameters was higher in patients who were treated with SIRT and sorafenib.

## Introduction

Hepatocellular carcinoma (HCC) is the fifth most common malignant tumour disease in the world.^1^ Cross‐sectional imaging, especially computed tomography (CT) and magnetic resonance imaging (MRI), plays an essential role in the diagnosis and local staging of HCC. Moreover, imaging can also provide data regarding tumour behaviour and prognosis.

For example, skeletal muscle condition has been identified as an important factor in patients with HCC in the study by Chang et al. (2018).^2^ Their study also showed that low skeletal muscle mass (LSMM) could predict relevant outcomes in patients with HCC.^2^ For instance, LSMM was associated with all‐cause mortality in patients with HCC (crude hazard ratio [HR] = 2.04, 95% confidence interval [CI]: 1.74–2.38; adjusted HR = 1.95, 95% CI: 1.60–2.37).^2^ LSMM can also predict lower objective response rate (odds ratio [OR] = 0.37, 95% CI: 0.17–0.81, *P* = 0.012) and more drug‐related adverse events (OR = 2.23, 95% CI: 1.17–4.28, *P* = 0.015).^3^ In addition, adipose tissue (AT), especially visceral adipose tissue (VAT), plays an important role in HCC. For instance, it was reported that preoperative visceral adiposity predicted poor outcomes after hepatectomy in patients with HCC.^4^ High VAT area reportedly affects survival in patients with advanced HCC treated with tyrosine kinase inhibitors.^5^

Some recent reports indicate that modern analysis of radiological images can provide more information about tissue composition. So far, radiomics is a modern analysis technique that quantitatively extracts features, including shape, size, intensity and texture of analysed tissue.^6^, ^7^, ^8^ According to the literature, radiomics parameters quantitatively visualize the heterogeneity of analysed tissues and reflect underlying pathophysiological changes.^9^, ^10^, ^11^ Radiomics parameters can predict tumour behaviour and prognosis, particularly in HCC.^10^ Presumably, radiomics features of body compartments like skeletal muscles and AT may be more sensitive for prediction of unfavourable prognosis in HCC in comparison with conventional analysis of body composition.

The purpose of the presented work was to investigate a possible predictive role of radiomics‐based body composition parameters in patients with HCC undergoing palliative treatment.

## Material and methods

### Patient data

This is a sub‐study of the SORAMIC trial, a prospective, randomized‐controlled, phase II trial performed at 38 sites in 12 countries in Europe and Turkey.^12^ The present study was performed within the palliative part of SORAMIC, where patients were randomized to receive sorafenib monotherapy or selective internal radiation therapy (SIRT) and sorafenib.^12^ In short, patients were eligible if they had preserved liver function (Child‐Pugh ≤ B7), an Eastern Cooperative Oncology Group performance status (ECOG PS) ≤ 2 and unresectable tumours not eligible for curative treatment or transarterial chemoembolization (TACE). For this sub‐study, a post hoc analysis of the prospective trial was conducted and the endpoint was the 1‐year survival.

Overall, there were 422 patients involved in the palliative part of SORAMIC. In 53 patients, no CT images were available in our institution and they were excluded from the present analysis. Furthermore, 72 patients were excluded because image artefacts or low image quality hindered the subsequent image segmentation. Therefore, the final cohort comprised 297 patients. There were 38 women (12.8%) and 259 men (87.2%) with a mean age of 67.0 ± 8.1 years, median age of 67 years, ranging from 46 to 85 years. After 1 year, 139 patients were alive and 158 deceased. We created two subgroups: Subgroup 1 comprises 147 patients with sorafenib monotherapy, and Subgroup 2 comprises only patients receiving sorafenib and SIRT (*n* = 150). Baseline patient characteristics are summarized in *Table* *1*.

**Table 1:** Clinical baseline characteristics of the two subgroups

Characteristics	Subgroup 1 Sorafenib (*n* = 147)	Subgroup 2 Sorafenib + SIRT (*n* = 150)
**Age, years, median (range)**	68 (46–85)	67 (50–84)
**Male/female (%)**	86.4/13.6	88.0/12.0
**BCLC stage (%)**		
A	1.3	3.3
B	32.0	31.3
C	66.7	65.4
**Aetiology, *n* (%)**		
AIH	1 (0.7)	0 (0)
Alcohol	54 (36.7)	59 (39.3)
Alcohol + viral	4 (2.7)	10 (6.7)
HBV	13 (8.8)	11 (7.3)
HCV	29 (19.7)	29 (19.3)
HC	1 (0.7)	6 (4.0)
NAFLD	10 (6.8)	6 (4.0)
NASH	8 (5.4)	13 (8.7)
NS	4 (2.7)	3 (2.0)
Cryptogenic	23 (15.6)	13 (8.7)
NAT	0	1 (0.7)
**ECOG (%)**	0: 73.4	0: 69.3
	1: 25.9	1: 28.7
	2: 0.7	C: 2.0

Abbreviations: AIH, autoimmune hepatitis; BCLC, Barcelona Clinic Liver Cancer; ECOG, Eastern Cooperative Oncology Group; HBV, hepatitis B virus; HC, haemochromatosis; HCV, hepatitis C virus; NAFLD, non‐alcoholic fatty liver disease; NASH, non‐alcoholic steatohepatitis; NAT, non‐alcoholic toxic; NS, not specified; SIRT, selective internal radiation therapy.

### Methods

**Figure 1:**
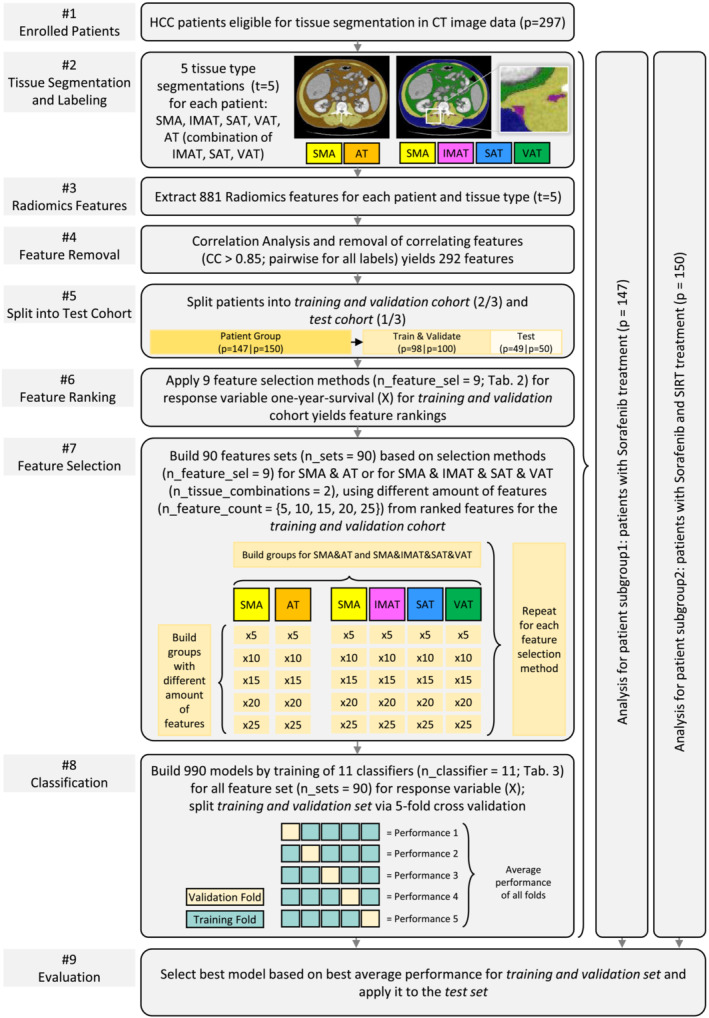
Pipeline illustrating our workflow. AT, adipose tissue subdivided into intramuscular adipose tissue (IMAT), subcutaneous adipose tissue (SAT) and visceral adipose tissue (VAT); CC, correlation coefficient; CT, computed tomography; HCC, hepatocellular carcinoma; SIRT, selective internal radiation therapy; SMA, skeletal muscle area.

In the following, we describe the preprocessing of the medical image data, the radiomics‐based feature extraction, the selection process to build various feature sets and the training of classifiers. The proposed workflow is illustrated in *Figure* *1*.

#### Tissue segmentation

**Figure 2:**
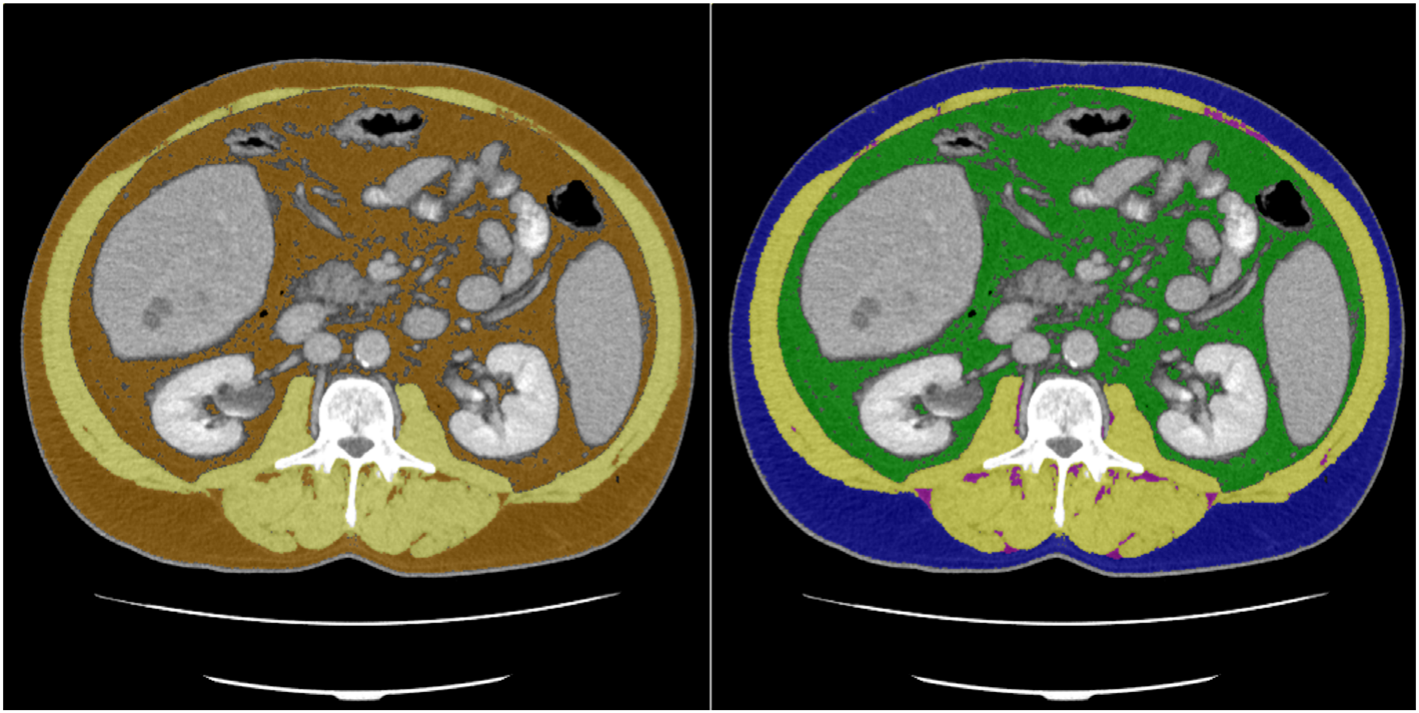
Labelled ground truth data with skeletal muscle mass (SMA) and adipose tissue (left) and SMA, intramuscular adipose tissue, subcutaneous adipose tissue and visceral adipose tissue (right).

Based on the CT image data, skeletal muscle area (SMA) and AT were segmented at the height of the L3 vertebrae (see *Figure* *2*). The segmentations were obtained semi‐automatically with the freely available ImageJ software 1.48v (National Institutes of Health Image programme). For further analysis, axial CT images at the L3 level in the soft tissue window (window, 45–250 HU) during portal venous phase were used. The segmentation comprised four tissue types: the SMA, which covers the musculus rectus abdominis, abdominal wall muscles, musculus psoas major, musculus quadratus lumborum and musculus erector spinae, as well as the AT subdivided into intramuscular adipose tissue (IMAT), subcutaneous adipose tissue (SAT) and VAT. For prognostic evaluation of radiomics analysis, we analysed two combinations: SMA and AT, as well as SMA and SAT, IMAT and VAT.

#### Radiomic feature extraction

For extraction of radiomic features, we used the pyradiomics library (https://pyradiomics.readthedocs.io/en/latest/), which is a Python‐based implementation,^13^ and applied it to the CT data. All datasets were normalized to −1024 and 1000 HU following the pyradiomics normalization steps. Tissue labels were created from the medical expert's segmentation. We used the settings that were recommended for CT data and automatically extracted 881 features for each of the four segmented tissue types (SMA, IMAT, SAT and VAT), as well as all fatty tissue labels combined for the fifth tissue type (AT). The results were stored in a large Excel file for subsequent analysis with MATLAB 2021a (The MathWorks, Natick, MA, USA; www.mathworks.com).

#### Feature cleansing

A correlation analysis was performed via MATLAB. In order to account for non‐normal features, we opted for Spearman's correlation instead of Pearson's correlation. We removed correlating features with correlation larger than 0.85 if the correlated feature pair was present in all tissue types. The first feature of the feature pair is removed, and the second one is kept until no correlating features exist. Feature cleansing reduced the feature number to 292 features for each patient and each tissue type.

#### Feature selection

For feature ranking, we applied nine feature selection methods, which are listed in *Table* *2*. The classifiers comprise infinite latent feature selection (ILFS),^14^, ^15^ feature selection via eigenvector centrality (ECFS), ReliefF algorithm, mutual information (MI), laplacian, fisher score, dependence‐guided unsupervised feature selection (DGUFS), unsupervised feature selection with ordinal locality (UFSOL) and least absolute shrinkage and selection operator (LASSO).^16^ We utilized the MATLAB framework Feature Selection Code Library (FSLib) for this step.^14^, ^15^, ^16^

**Table 2:** Feature selection methods for extraction of feature ranking

Number	Abbreviation	Algorithm
1	ILFS	Infinite latent feature selection
2	ECFS	Feature selection via eigenvector centrality
3	ReliefF	ReliefF
4	MI	Mutual information
5	laplacian	Laplacian
6	fisher	Fisher's score
7	DGUFS	Dependence‐guided unsupervised feature selection
8	UFSOL	Unsupervised feature selection with ordinal locality
9	LASSO	Least absolute shrinkage and selection operator

Afterwards, we built feature sets using different feature set sizes (n_feature_count = {5,10,15,20,25}). A feature count of 5 means that the 5 highest ranked features (based on one of the feature ranking algorithms) are selected to build a feature set. We opted for the two tissue subdivisions:

1. extracting features for SMA and AT; and
2. extracting features for SMA, IMAT, SAT and VAT.

For example, applying a feature count of 5, this results in 10 features (5 from SMA and 5 from AT) for the first option and 20 features (5 from SMA, 5 from IMAT, 5 from SAT and 5 from VAT) for the second option. Using the different feature counts, we obtain 5 combinations for the 2 options and the 9 feature selection methods yielding a total of 90 feature sets. The feature sets are also illustrated in *Figure* *1*, Step #7 Feature Selection.

#### Training of classifiers

For the classification based on the ranked feature sets, we trained 11 classifiers, similar to a previous study,^17^ but added linear logistic regression (LogReg). The classifiers are listed in *Table* *3*. The classifiers Adaptive boosting classifier (ADAC), bagging classifier (BAGC), decision tree classifier (DTC), K nearest neighbourhood classifier (KNNC), random forest classifier (RFC), support vector machine classifier (SVMC) and LogReg were implemented via MATLAB's Statistics and Machine Learning Toolbox. For Bernoulli Naïve Bayesian (BNB), stochastic gradient descent classifier (SGDC) and extreme gradient boosting classifier (XGBC), the publicly available MATLAB third‐party toolboxes were adapted to our requirements and integrated in our workflow.^18^, ^19^, ^20^

**Table 3:** Machine learning algorithms for 1‐year survival classification

Number	Abbreviation	Algorithm
1	ADAC	Adaptive boosting classifier
2	BAGC	Bagging classifier
3	BNB	Bernoulli Naïve Bayesian
4	DTC	Decision tree classifier
5	GNBC	Gaussian Naïve Bayesian classifier
6	KNNC	K nearest neighbourhood classifier
7	RFC	Random forest classifier
8	SGDC	Stochastic gradient descent classifier
9	SVMC	Support vector machine classifier
10	XGBC	Extreme gradient boosting classifier
11	LogReg	Linear logistic regression

As illustrated in *Figure* *1*, each classifier was trained for the 90 feature sets yielding 990 trained models. We split each patient group into a training and validation set, comprising two thirds of the patients, and a test set, comprising one third of the patients. Performance for training and validation was measured based on five‐fold cross‐validation and averaging over the individual folds' accuracies.

Next, we evaluated the classifiers' performance for the test sets for Subgroup 1, that is, patients who underwent sorafenib monotherapy, and Subgroup 2, that is, patients who underwent SIRT and sorafenib treatment.

## Results

**Figure 3:**
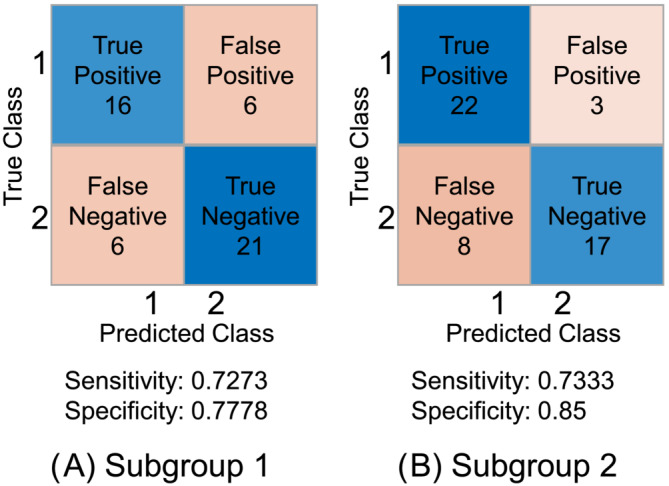
(A, B) Depiction of class versus prediction including true positive, false positive, false negative, true negative, sensitivity and specificity. Positive class (1) indicates that 1‐year survival is positive, that is, the patients live, and negative class (2) indicates that 1‐year survival is negative, that is, the patients are deceased.

We applied the presented pipeline to 297 patients, where 139 patients were alive and 158 deceased after 1 year. Subgroup 1 comprises 147 patients with sorafenib monotherapy, and Subgroup 2 comprises only patients (*n* = 150) receiving sorafenib and SIRT. For the trained models, linear logistic regression performed best for the subgroup treated with sorafenib with an accuracy of 75.51%. The accuracy was 78.00% for the subgroup treated with SIRT and sorafenib based on the ADAC classifier. True positive (TP), true negative (TN), false positive (FP) and false negative (FN) values are illustrated in *Figure* *3*.

**Figure 4:**
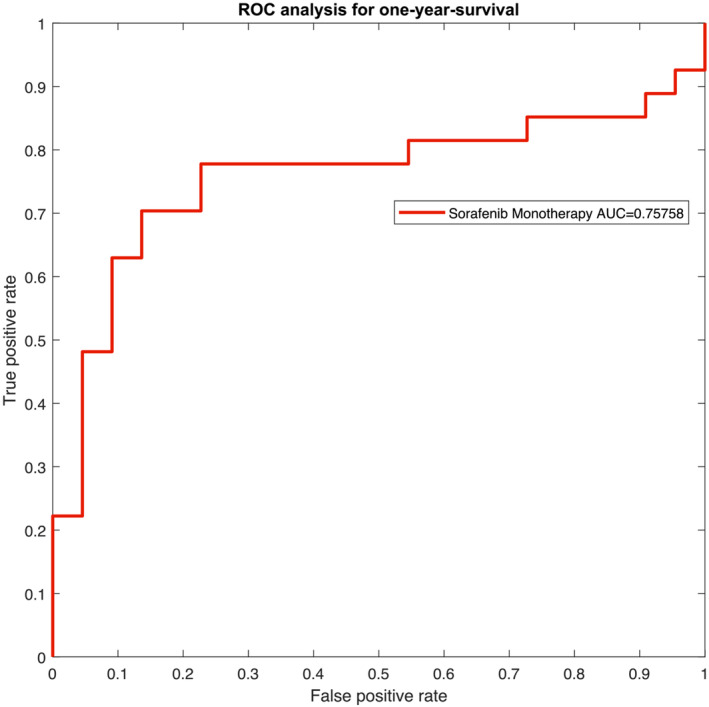
Depiction of the receiver operating characteristic (ROC) curve for Subgroup 1, that is, patients who underwent sorafenib monotherapy. AUC, area under the curve.

**Figure 5:**
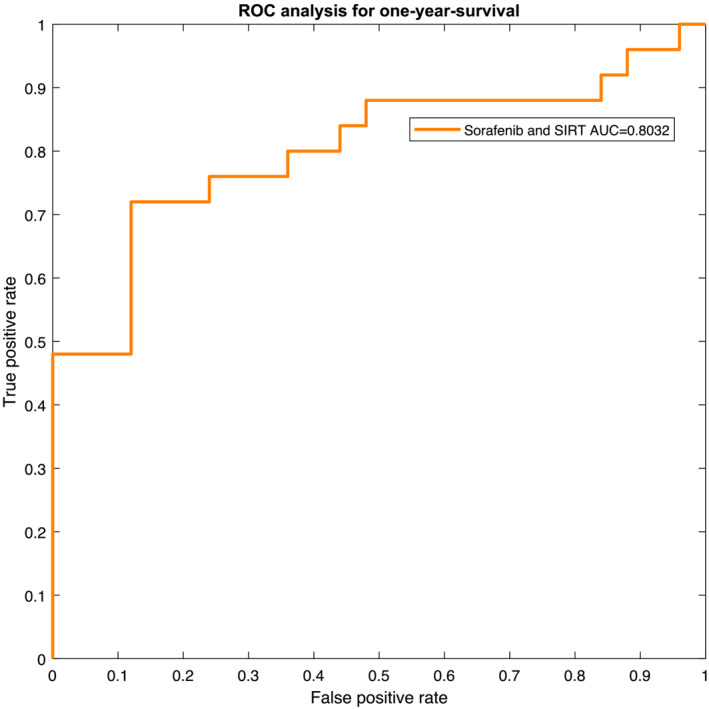
Depiction of the receiver operating characteristic (ROC) curve for Subgroup 2, that is, patients who underwent selective internal radiation therapy (SIRT) and sorafenib treatment. AUC, area under the curve.

The resulting receiver operating characteristic (ROC) curves for the two patient groups are presented in *Figures* *4* and *5*. The ROC yields an area‐under‐the‐curve (AUC) value of 0.7576 with a 95% CI of 0.6376–0.8776 for Subgroup 1. For Subgroup 2, AUC was 0.8032 (95% CI: 0.6930–0.9134).

For all the 990 trained models, the best classification result was achieved by a feature set comprising SMA and AT and applying the ReliefF feature selection method.

## Discussion

As already mentioned, parameters of body composition are of prognostic significance in HCC. So far, in curative setting (hepatectomy), LSMM predicted lower overall survival (OS) (HR = 2.17, 95% CI: 1.48–3.19, *P* < 0.00001).^21^ Interestingly, LSMM was also associated with lower recurrence‐free survival after tumour resection (HR = 1.79, 95% CI: 1.28–2.50, *P* < 0.00001).^21^ Furthermore, in palliative setting, sarcopenic patients with HCC treated with kinase inhibitors like sorafenib or lenvatinib showed lower OS (HR = 2.24, 95% CI: 1.60–3.14, *P* < 0.00001) than patients without sarcopenia.^21^

Also, AT plays an important role in HCC. Patients with high subcutaneous adipose tissue index (SATI) had significantly better progression‐free survival (*P* = 0.0093) and OS (*P* = 0.032) than those with low SATI.^22^ In contrast, patients with high VAT had shorter OS (HR = 1.35, 95% CI: 1.09–1.66, *P* < 0.005).^23^ Finally, high VAT to SAT ratio also predicted negative OS (HR = 1.57, 95% CI: 1.22–2.01, *P* < 0.001).^4^

Importantly, the quality of the skeletal musculature and AT also plays a significant role. In fact, tumour patients with low density of the skeletal musculature had a 75% higher mortality risk than patients with high or normal muscle density (HR = 1.75, 95% CI: 1.60–1.92, *P* < 0.00001).^24^ Moreover, in HCC, low skeletal muscle density was stronger associated with mortality risk (HR = 1.88, 95% CI: 1.40–2.52, *P* < 0.00001).^24^ Furthermore, high density of SAT and VAT correlated negatively with survival in patients with HCC.^25^, ^26^

Therefore, a deep analysis of the quality of the skeletal musculature and AT may provide novel relevant parameters that can have predictive values in HCC. Our results confirm this hypothesis. The present work is the first report about the prognostic role of radiomics parameters of body composition compartments in HCC. As shown in the presented study, radiomics features of the skeletal musculature and AT can predict 1‐year survival, with an accuracy value of 75.51% (AUC = 0.7576, 95% CI: 0.6376–0.8776) for patients who underwent sorafenib monotherapy and an accuracy value of 78.00% (AUC = 0.8032, 95% CI: 0.6930–0.9134) for patients who underwent treatment with SIRT and sorafenib.

Our values for the radiomics‐based analysis of the two subgroups are superior to those reported for the conventional analysis of body composition. For example, Hou et al.^27^ showed that sarcopenia can predict 1‐year survival in HCC with an AUC value of 0.7. In addition, there was no relevant association of body composition parameters with OS either in the sorafenib monotherapy subgroup or in the sorafenib and SIRT treatment subgroup.^28^

The identified significant influence of the quality of the skeletal muscles and fat investigated by radiomics‐based parameters on survival in patients with HCC is multifactorial.^29^ The skeletal musculature significantly influences the immune system.^30^ For instance, skeletal muscles produce several cytokines (myokines) with immune effects.^31^ So far, interleukin (IL)‐15 is a myokine that stimulates proliferation and activation of natural killer (NK) cells and CD8+ T lymphocytes, which have an important antitumoural effect.^32^ Interestingly, intravenous administration of IL‐15 induced a significant increase of circulating CD8+ T and NK cells in patients with different tumours.^33^ Presumably, reduced and/or altered musculature may synthesize a smaller number of myokines. AT also plays an important role in immune anticancer activity. VAT secrets proinflammatory cytokines tumour necrosis factor‐α and IL‐6.^34^ Furthermore, VAT correlated with circulating leptin level.^35^ Leptin promotes the growth and proliferation of tumour cells by activating various signalling pathways.^36^ In HCC, leptin promotes invasion and migration of HCC cells.^37^

We hypothesize that texture analysis parameters of the skeletal musculature and AT may reflect deep changes and metabolic activity of the tissues.

Our study has several limitations. The manual segmentation of SMA, IMAT, SAT and VAT is error‐prone and could be improved by additional readers. However, as the radiomics features are due to structural or textural peculiarities, the influence of smaller components or small variations within the segmentation is considered to be very small. Future work can replace the manual segmentations with automatic ones.^38^, ^39^

In addition, although the present data are based on a multicentre cohort, the postprocessing was performed unicentric, that is, a single university hospital. Therefore, we subdivided the data into a test cohort and a training and validation cohort. In future work, we plan to extend our approach to a multicentre study, as conducted by Feng et al.^40^

In conclusion, parameters of radiomics‐based analysis of the skeletal musculature and AT predict 1‐year survival in patients with advanced HCC. The prognostic value of radiomics‐based parameters was higher in patients who were treated with SIRT and sorafenib.

## Conflict of interest statement

The authors declare that there are no conflicts of interest.
